# Dietary fiber-based regulation of bile salt hydrolase activity in the gut microbiota and its relevance to human disease

**DOI:** 10.1080/19490976.2022.2083417

**Published:** 2022-06-05

**Authors:** Arthur Kastl, Wenjing Zong, Victoria M. Gershuni, Elliot S. Friedman, Ceylan Tanes, Adoma Boateng, William J. Mitchell, Kathleen O’Connor, Kyle Bittinger, Natalie A. Terry, Christina Bales, Lindsey Albenberg, Gary D. Wu

**Affiliations:** a Division of Gastroenterology, Hepatology, and Nutrition, the Children’s Hospital of Philadelphia, Philadelphia, PA, USA; b Department of Surgery, Perelman School of Medicine, University of Pennsylvania, Philadelphia, PA, USA; c Division of Gastroenterology and Hepatology, Perelman School of Medicine, University of Pennsylvania, Philadelphia, PA, USA

**Keywords:** Short bowel syndrome, parenteral nutrition, microbiota, bile acids

## Abstract

Complications of short bowel syndrome (SBS) include malabsorption and bacterial overgrowth, requiring prolonged dependence on parenteral nutrition (PN). We hypothesized that the intolerance of whole food in some SBS patients might be due to the effect of dietary fiber on the gut microbiome. Shotgun metagenomic sequencing and targeted metabolomics were performed using biospecimens collected from 55 children with SBS and a murine dietary fiber model. Bioinformatic analyses were performed on these datasets as well as from a healthy human dietary intervention study. Compared to healthy controls, the gut microbiota in SBS had lower diversity and increased Proteobacteria, a pattern most pronounced in children on PN and inversely correlated with whole food consumption. Whole food intake correlated with increased glycoside hydrolases (GH) and bile salt hydrolases (BSH) with reduced fecal conjugated bile acids suggesting that dietary fiber regulates BSH activity via GHs. Mechanistic evidence supporting this notion was generated via fecal and plasma bile acid profiling in a healthy human fiber-free dietary intervention study as well as in a dietary fiber mouse experiment. Gaussian mixture modeling of fecal bile acids was used to identify three clinically relevant SBS phenotypes. Dietary fiber is associated with bile acid deconjugation likely via an interaction between gut microbiota BSHs and GHs in the small intestine, which may lead to whole food intolerance in patients with SBS. This mechanism not only has potential utility in clinical phenotyping and targeted therapeutics in SBS based on bile acid metabolism but may have relevance to other intestinal disease states.

## Introduction

Bile acids play a fundamentally important role in nutrient absorption through the emulsification and solubilization of fat in the small intestine. Additionally, they regulate mammalian physiology via activation of nuclear hormone and G-protein coupled receptors.^1^ Amino acid conjugation of bile acids to either glycine or taurine increases their solubility and reduces epithelial passive absorption, thereby preserving high levels of functional bile acids in the small intestine.^2^ Bacterial bile salt hydrolases (BSHs) are responsible for the rate-limiting step of bile acid deconjugation which is followed by conversion of primary into secondary bile acids via bacterial dehydroxylation.^3^ While these processes occur normally in the colon, deconjugation of bile acids may occur abnormally in the small intestine. This is clinically relevant in patients with small intestinal bacterial overgrowth (SIBO), where a high deconjugated bile acid load may lead to reduced nutrient absorption and lipid malabsorption.^4^ SIBO occurs when there is an abnormally high biomass of the gut microbiota in the small intestine which may lead to altered bile acid profile, carbohydrate fermentation, nutrient competition, and epithelial dysfunction. The pathophysiologic effects of SIBO are particularly deleterious in patients with short bowel syndrome (SBS) who are already at risk for poor nutrient assimilation.

SBS is a malabsorptive state, involving insufficient bowel length to maintain protein-energy, fluid, electrolyte, and micronutrient balances while on an enteral diet.^5^ Following substantial bowel loss, the remaining intestine adapts by increasing its innate absorptive capacity and length, and the majority of children eventually progress to enteral autonomy.^6^ Children with SBS require parenteral nutrition (PN) for months to years to support nutrition and growth, and this intervention often contributes to morbidity via recurrent central-line infections, thrombosis, and hepatic injury.^7^ Thus, clinicians place strong emphasis on maximizing enteral nutrition, particularly whole food consumption, to the extent tolerated. However, tolerance of whole food consumption can be limited by gas distension, early satiety, vomiting, excessive diarrhea, and other symptoms suggestive of bacterial overgrowth, a complication which often increases reliance on PN. Given that there are inherent limitations to hydrogen breath testing and duodenal aspirate analysis in children with SBS to identify SIBO, it is common clinical practice (in the absence of clear guidelines) to empirically treat with antibiotics in these instances, which ameliorates symptoms in some children, and thus arguing for a bacterial contribution to this clinical picture. Small studies have demonstrated that patients with SBS have intestinal dysbiosis, an alteration in the composition and/or biomass of the microbiota generally associated with a reduction in diversity, and a predisposition for SIBO.^6^ Adults with SBS have been found to have reduced microbial diversity in feces as well as altered plasma and fecal bile acid profiles compared with healthy controls, whereas patients weaned from PN showed an increase in secondary fecal bile acid levels.^8^ Currently, decisions about dietary interventions in patients with SBS are largely empiric due to a lack of clinical phenotyping tools that can predict outcomes and/or guide therapeutic interventions to promote dietary tolerance of whole foods.

Whole food diets will contain fiber which has been shown to have a significant effect on the composition and function of the gut microbiota, as well as metabolite production, in murine model systems where robust effects on host physiology are observed.^9–11^ By contrast, the effects of dietary fiber are less robust in humans in part, due to high levels of intersubject variability.^12–14^

By performing an integrative analysis of diet, the microbiome, and bile acid profiling in 55 pediatric patients with SBS, we provide evidence supporting the notion that dietary fiber regulates BSH activity via GHs. These alterations can also be detected in the plasma of patients. Studies in a mouse model, as well as in a dietary intervention study in healthy human subjects, provide mechanistic evidence linking the consumption of dietary fiber with BSH activity through bacterial glycoside hydrolases (GH) specifically in the small intestine. This evidence provides an explanation for the association between symptoms associated with SIBO and the increased plasma levels of unconjugated bile acids, which may serve as a biomarker for patients with SBS who may be at risk for malabsorption upon the introduction of a whole food diet. These findings demonstrate the potential value of the gut microbiome and its co-metabolites as a prognostic platform for precision nutrition in this population. Indeed, using Gaussian mixture modeling, we identified three fecal bile acid patterns that correlated with clinical phenotypes and may have utility in both predicting tolerance to the introduction of whole foods, as well as guiding targeted therapeutic interventions to enhance dietary intake and nutrient assimilation.

## Results

### Clinical characteristics of the SBS study cohort

Fifty-five participants with SBS were enrolled, with a median age of 6.7 years (range 9 months to 17 years). The most common diagnoses leading to bowel resection were necrotizing enterocolitis, gastroschisis, and bowel atresia (Table 1), representative of the pediatric SBS population at large. Anatomic characteristics of these patients, including bowel anastomotic site and remaining small bowel length (cm), are also provided. Thirty-eight percent of the study cohort were on some amount of PN at enrollment. While some participants were nearly exclusively PN-dependent, formula-fed EN, or consuming whole foods, the majority received some combination thereof (Supplemental Figure 1). Additionally, 30% of the study participants were on antibiotic therapy for presumed SIBO at the time of sample collection (Table S1). Based on sample availability, 46 fecal samples were analyzed for microbiome composition, 45 fecal samples were analyzed for bile acid composition, and 35 plasma samples were analyzed for bile acid composition and FGF19 levels.

**Table 1.** Clinical characteristics of patients with short bowel syndrome (SBS)

Variable	Value
Total patients, No.	55
Male, No. (%)	30 (54)
Age at enrollment, median (range), y	6.7 (0.75–17)
Diagnosis resulting in SBS, No.	
Necrotizing enterocolitis (%)	21 (38)
Gastroschisis (%)	13 (24)
Atresia (%)	11 (20)
Volvulus (%)	5 (9)
Other (OEIS, internal hernia) (%)	5 (9)
Anastomosis location, No.	
Jejunocolic (%)	16 (29)
Ileocolic (%)	19 (35)
Small bowel-small bowel (%)	15 (27)
Small bowel ostomy (%)	5 (9)
Remaining small bowel length, mean (range), cm	52 (10–150)
Receiving PN, No. (%)	21 (38)
Receiving antibiotic therapy for SIBO, No. (%)	16 (30)

OEIS, omphalocele-exstrophy-imperforate anus syndrome; PN, parenteral nutrition; SBS, short bowel syndrome

### The consumption of whole food is associated with greater gut microbiota diversity and a different taxonomic composition, with higher levels of Bacteroides spp. and a reduction in Enterobacteriaceae

**Figure 1.**
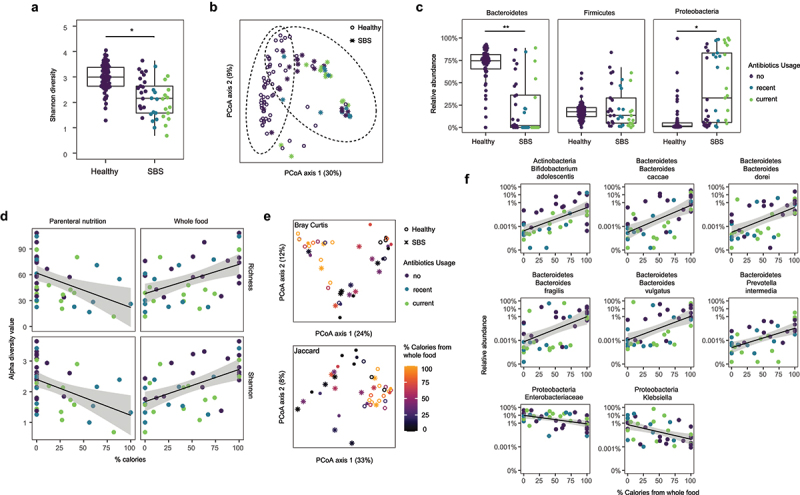
Impact of parenteral nutrition (PN) vs. a whole food diet on the composition of the gut microbiota in patients with SBS after controlling for antibiotic use. (a) difference in Shannon diversity of the gut microbiota between healthy controls vs. SBS (p = .018, linear model with antibiotics usage as a covariate). (b) principal component analysis beta-diversity in the feces of SBS vs. healthy cohorts using bray-curtis distances (p < .001, PERMANOVA). (c) Relative abundance of bacteroidetes, proteobacteria, and firmicutes in fecal samples of healthy controls vs. patients with SBS (fdr = 0.0057, 0.38 and 0.016, respectively). (d) richness and Shannon diversity of the fecal gut microbiota correlated with %PN (p = .033 and 0.0048, respectively) and %whole food intake (p = .052 and 0.0025, respectively, fixed-effect linear models with antibiotics use and antibiotics type as covariates). (e) bray-curtis and (f) jaccard principal component analysis of beta-diversity correlated with %whole food intake as indicated in the color-coded index (PERMANOVA p = .002 and 0.015, respectively). (g) Positive correlations of %calories from whole food consumption with the relative abundance *bifidobacterium adolescentis, bacteroides caccae, bacteroides dorei, bacteroides fragilis, bacteroides vulgatus, prevotella intermedia* (fdr = 0.0081, 0.0081, 0.0081, 0.012, 0.012, and 0.093, respectively; fixed effects linear modeling). negatively correlated bacterial taxa include two proteobacterial taxa, *enterobacteriaceae and klebsiella* (fdr = 0.019 for both; fixed effects linear models). in all panels, *p or q < 0.05; **p or q < 0.01, and; ***p or q < 0.001.

The gut microbiome in children with SBS had reduced alpha-diversity relative to healthy controls (Figure 1a). The overall composition of the gut microbiota was different between SBS and healthy controls based on beta-diversity (Figure 1b). At the phylum level, this difference in composition was due to a decrease in Bacteroidetes and an increase in Proteobacteria in SBS (Figure 1c). These findings, which were independent of antibiotic use, corroborate other smaller studies in adults and children with SBS,^15^ where the decrease in diversity and predominance of Proteobacteria was associated with remnant bowel length^16^ and poor growth.^17^

Previous studies have emphasized the importance of diet on the composition of the human gut microbiota, where fiber plays a particularly important role in both mice and humans.^9,10,18^ The diverse nature of dietary intake in the SBS population, ranging from PN to formula EN and whole food in varying proportions, provided an opportunity to characterize the effect of diet on the composition of the gut microbiota in SBS. The two dietary extremes in our cohort were exclusive PN and the exclusive consumption of whole food. PN vs. whole food intake led to a reduction in alpha-diversity as quantified by both richness and Shannon diversity (Figure 1d). Moreover, the composition of the gut microbiota in fecal samples was distinctly different in those children with SBS consuming a higher proportion of whole food (greater than 80%) by Bray-Curtis and Jaccard beta-diversity, respectively (Figure 1e). The consumption of whole food in children with SBS was negatively correlated with Enterobacteriacea, specifically *Klebsiella spp*, and positively correlated with a number of bacterial taxa belonging to *Bacteroides, Prevotella*, and *Bifidobacteria* generae, known for their ability to digest complex glycans found in dietary fiber (figure 1f).^19^ Indeed, using mixed model linear regression in this SBS cohort, we found that only the consumption of whole food (p = .0064) but not remnant small bowel length, presence/absence of the ileocecal valve, or antibiotic exposure, was statistically correlated with microbiota composition.

### Glycoside hydrolase and bile salt hydrolase gene abundance is correlated with whole food intake

**Figure 2.**
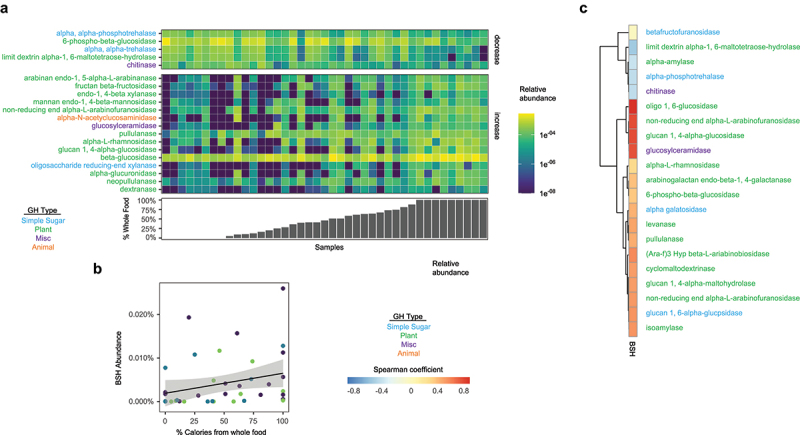
Correlations between %whole food intake and the relative abundance of glycoside hydrolases (GH) and bile salt hydrolases (BSH) in the gut microbiome of patients with SBS. (a) The significantly correlated relative abundance of twenty GHs with %calories from whole food using mixed-effect linear modeling (fdr<0.05) (b) The relative abundance of BSH genes correlated with %calories from whole food (q = 0.035, fixed-effects linear modeling with antibiotics use as a covariate). (c) Correlations between GH and BSH gene abundances (Spearman coefficients for genes with statistically significant correlations based on simple linear regression with fdr<0.05). Known general substrates for GHs are provided in colored text.

Glycoside hydrolases (GH) are a broad family of enzymes involved in degradation of starch, cellulose, chitin, and other carbohydrates. Although eukaryotic cells contain GHs, the bacterial contribution far exceeds what a human host epithelium expresses.^19^ *Bacteroides spp*. are especially well equipped to digest complex glycans abundant in dietary fiber, whereas bacterial taxa belonging to the Proteobacteria phylum generally have lower ability to digest fiber, instead relying on simple carbohydrates as a carbon source.^9^ In our SBS cohort, we found a strong positive association between the percentage of whole food consumption and GH gene abundance focused on primarily plant glycan degradation (Figure 2a).

Biochemical mechanisms important in food digestion, such as the emulsification of dietary fat by bile acids, are vital in patients with SBS due to the reduction of small intestinal surface area needed for nutrient absorption. Gut bacteria are crucial modifiers of luminal bile acids. Many bacteria have bile salt hydrolases (BSHs), enzymes which deconjugate the glycine or taurine amino acid from bile acids.^20^ The conversion from primary bile acids to secondary via 7a-dehydroxylation is accomplished by only a few bacteria taxa that have BAI operons, and all of these taxa belong to Clostridium and Eubacterium.^21^ The amino acid conjugation of bile acids plays a critical role in maintaining their optimal digestive activity in the small bowel, in part by increasing their aqueous solubility. This conjugation limits bile acid absorption and helps maintain high levels in the small intestine to aid in digestion. The deconjugation of bile acids by BSHs in the small intestine would, therefore, reduce digestive efficiency, and increase the risk for fat malabsorption, a particular concern in patients with SBS. In addition to GHs, whole food intake was positively correlated with BSH gene abundance in SBS (Figure 2b). 21 GHs were significantly correlated with BSHs in the SBS cohort, the majority showing positive correlations with GHs involved in plant-based glycan metabolism (Figure 2c), suggesting the role of dietary fiber in BSH gene abundance in the gut microbiome of children with SBS. In total, these results are consistent with the notion that consumption of whole foods may promote growth of bacteria that metabolize complex glycans in dietary fiber and that have abundant BSHs, thereby increasing the deconjugation of primary bile acids in the small intestine.

### Statistical modeling identifies three fecal bile acid patterns that are associated with clinical phenotypes in patients with SBS

**Figure 3.**
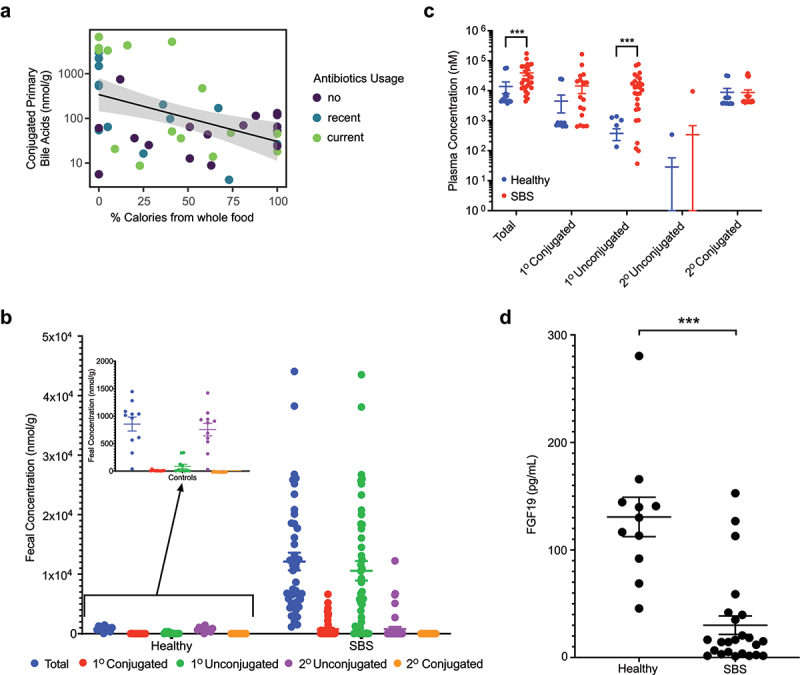
Comparison of fecal and plasma bile acid profiles between patients with SBS and healthy controls. (a) correlation between conjugated fecal bile acids with %calories from whole food intake in patients with SBS (q = 0.041; fixed effects modeling with antibiotic use as a covariate). (b) fecal bile acid profiles in patients with SBS and healthy controls. (c) plasma bile acid levels in patients with SBS vs. healthy controls (fdr = 0.0007 and 0.00005 for total and primary unconjugated bile acids, respectively; Mann-Whitney tests with FDR correction). (d) plasma FGF19 levels in patients with SBS vs. healthy controls (p < .0001, two-tailed unpaired Student’s t-test). In all panels, *p or fdr<0.05; **p or fdr<0.01, and; ***p or fdr<0.001.

To determine the functional consequences of the association between whole food intake and BSHs, we characterized the composition of fecal bile acids in patients with SBS relative to healthy children. Consistent with the positive correlation between the percentage of whole food intake and BSH gene representation, we found that fecal conjugated primary bile acids were negatively correlated with whole food intake (Figure 3a). Absolute quantification and conjugation status of fecal bile acids revealed a two-log increase in primary unconjugated bile acids in SBS compared to age-matched controls, which accounted for the vast majority of the fecal bile acids in most of the SBS cohort (Figure 3b and Supplemental Figure 2). The deconjugation of primary bile acids, normally found at high levels in the small intestine, would not only reduce their ability to emulsify dietary fat for digestion but would also increase their intestinal absorption, leading to higher levels in the systemic circulation.^2^ Indeed, children with SBS had high levels of total plasma bile acids, due mostly to an increase in unconjugated primary bile acids (Figure 3c).

**Figure 4.**
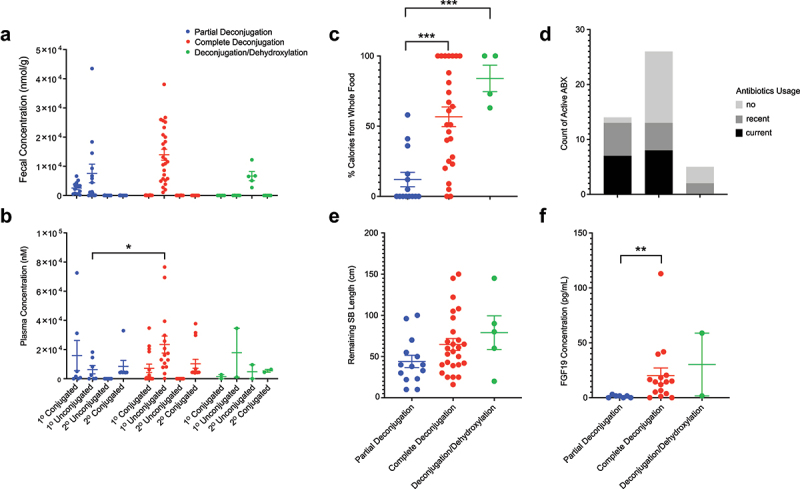
Gaussian Mixture modeling (GMM) based on fecal bile acid composition. (a) fecal bile acid levels in three bile acid composition groups. (b) composition of plasma bile acid levels based on three patient groups as defined by GMM (p = .03 for primary unconjugated bile acids between partial deconjugation and Complete deconjugation groups; Mann-Whitney test). characteristics of the three GMM patient classifications based on (c) %calories from whole food (p < .001 by one-way ANOVA; p < .0001 for Partial Deconjugation vs. Complete Deconjugation and p = .0001 for Partial Deconjugation vs. Deconjugation/Dehydroxylation by Fisher’s LSD); (d) Antibiotic use; (e) Remaining small bowel length (p = .1046, one-way ANOVA); and (f) Plasma FGF19 concentrations (p = .0213, Kruskal-Wallis test, and; p = .007 for Partial Deconjugation vs. Complete Deconjugation, Dunn’s test). In all panels, *p < .05; **p < .01, and; ***p < .001.

The alteration in fecal bile acid composition in patients with SBS led us to use Gaussian Mixture Modeling to determine if it was possible to classify patients into specific groups based on fecal bile acid patterns. This analysis identified three specific bile acid composition patterns based on levels of primary conjugated, primary unconjugated, or secondary unconjugated bile acids. The three patterns are shown in Figure 4a. The first is defined by high levels of primary conjugated bile acids (>200 nmol/g) as well as minimal amounts of secondary bile acids, suggesting incomplete deconjugation of conjugated bile acids by bacterial BSHs (termed the “Partial Deconjugation” pattern). The second is defined by a majority of primary unconjugated bile acids along with minimal amounts of primary conjugated and secondary bile acids, suggesting complete or near-complete deconjugation but no dehydroxylation (termed the “Complete Deconjugation” pattern). The third is defined by high levels of secondary bile acids along with minimal levels of primary conjugated or unconjugated bile acids, suggesting complete or near-complete deconjugation and dehydroxylation (termed the “Deconjugation/Dehydroxylation” pattern). The elevated plasma levels of primary unconjugated bile acids in patients with SBS relative to healthy controls (Figure 3c) is a primary feature of the “Complete Deconjugation” groups (Figure 4b).

In our study, 14 SBS patients had the partial deconjugation pattern, 26 had the complete deconjugation pattern, and 5 had the deconjugation/dehydroxylation pattern. Each bile acid pattern was associated with a different clinical phenotype associated with whole food intake and antibiotic use (Figures 4C-e, Table S1). Patients with the Partial Deconjugation pattern were consuming only a minimal amount of whole foods (12% ± 5%, mean ± SEM, total daily calories from whole foods) and 93% had current (50%) or recent (43%) antibiotic usage for SIBO. This group includes the 8 patients that were on greater than 50% PN (Supplemental Figure 1) where fecal levels of primary conjugated bile acids were much greater than the other two groups (Figure 4a). We calculated the ratio of primary conjugated bile acids to primary unconjugated bile acids, and indeed the ratio was much higher in these 8 patients (ratio = 0.39) than the rest of our cohort with PN consumption less than 50% (ratio = 0.05). Patients with the Complete Deconjugation pattern were consuming some whole foods (57% ± 7% total daily calories from whole foods) and 50% had current (31%) or recent (19%) antibiotic usage. Finally, all patients with the Deconjugation/Dehydroxylation pattern were consuming a substantial amount of whole food and/or dietary fiber (84% ± 8.5% total daily calories from whole foods), and only 40% had either current (0%) or recent (40%) antibiotic usage. Interestingly, remnant small bowel length was not correlated with these three patterns (Figure 4e).

The three fecal bile acid patterns are a manifestation of two general mechanisms by which the gut microbiota metabolize bile acids – deconjugation and dehydroxylation. Deconjugation of bile acids is performed by bile salt hydrolase (BSH) enzymes, and the BSH genes encoding for these enzymes are found in hundreds of bacterial strains known to inhabit the gastrointestinal tract of mammals.^22^ By contrast, the transformation of primary to secondary bile acids via 7α-dehydroxylation, which is performed by genes in the bile-acid-induced operon (termed Bai) containing eight genes, is only performed by a few select *Clostridium spp*.^23^ Although the entire eight-gene bai operon is found in all strains known to 7α-dehydroxylate, only the combination of BaiB, BaiCD, BaiA2, BaiE, BaiF, and BaiH is required for the reaction.^23^ While BaiA, BaiB, BaiCD, BaiF, and BaiH were found in nearly all samples in our dataset, BaiE, BaiG, and BaiI were only detected in five fecal samples (Supplemental Figure 3), all of which were in the Deconjugation/Dehydroxylation pattern that is most similar to the profile of healthy children (Figure 3b and Supplemental Figure 2).

Regardless of the bile acid pattern, total fecal bile acid levels in SBS patients were 1–2 logs greater than found in healthy controls (Figure 3b). The high levels of fecal bile acids in these patients are likely due to the reduction in ileal reabsorption of bile acids with the surgical resection of the distal small bowel in most of these patients. Resection of the terminal ileum would also lead to disruption of the negative feedback loop suppression of bile acid synthesis with reduced activation of the farnesoid X receptor (FXR), which is expressed at high levels in the intestinal epithelium of distal small bowel.^24^ Indeed, plasma fibroblast growth factor 19 (FGF19) levels, the mediator of FXR-dependent suppression of hepatic bile acid synthesis,^25^ was significantly lower in patients with SBS compared to healthy controls (Figure 3d) due to essentially undetectable levels in the Partial Deconjugation group (figure 4f).

The high levels of fecal unconjugated primary bile acids in patients with SBS compared to healthy controls (Figure 3b and Supplemental Figure 2) is an unusual pattern that provides an explanation for the lack of secondary bile acids in these patients. The high levels of fecal bile acids may prevent the growth of critical bacterial taxa with the full complement of Bai genes needed for the production of secondary bile acids (Supplemental Figure 3).^23^ Indeed, millimolar concentrations of bile acids observed in subjects with SBS have been shown to inhibit the growth of BAI operon containing bacterial species *in vitro*.^26^

### The absence of dietary fiber reduces BSH activity of the gut microbiome, thus preserving the abundance of conjugated fecal bile acids in healthy human subjects

**Figure 5.**
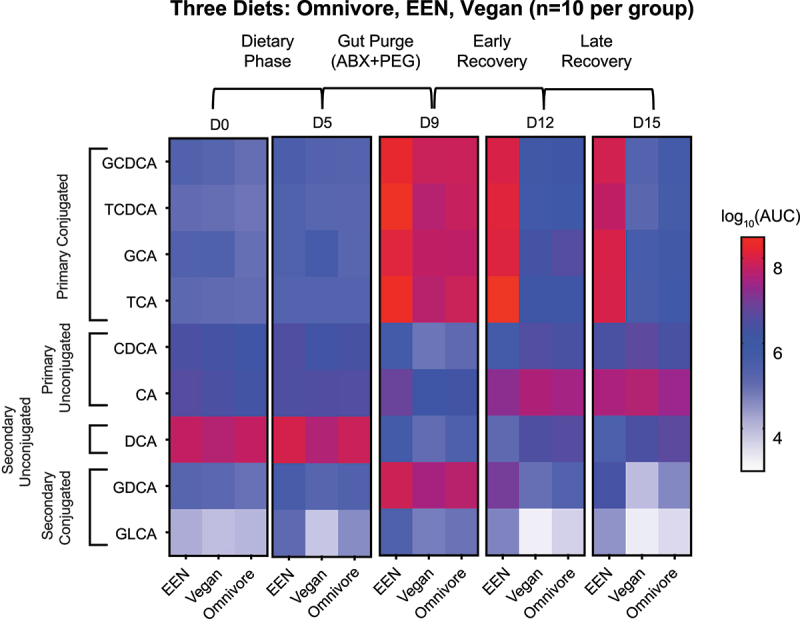
Fecal bile acid levels in healthy adults consuming exclusive enteral nutrition (EEN), vegan, or omnivore diets and receiving a gut purge (antibiotics and polyethylene glycol) between Days 5 and 9 in the FARMM study.^18^ Overall design of the study and bile acid levels in the feces of subjects on Days 0, 5, 9, 12, and 15 of the study. Bile acid levels are shown as log_10_-transformed area under the curve values.

To provide direct evidence that fiber was the active component of whole food responsible for the enhancement of BSH gene representation (Figure 2), thereby enhancing bile acid deconjugation in the gut lumen of SBS patients (Figure 3a), we quantified fecal bile acids in human adult subjects throughout the course of the Food and Resulting Microbial Products (FARMM) study.^18^ FARMM was a longitudinal domiciled dietary study examining the effect of omnivore, vegan, and fiber-free exclusive enteral nutrition (EEN) diets on the composition of the gut microbiome and metabolome before (days 1–5) and upon recovery after a gut purge of antibiotics and polyethylene glycol (days 9–15) (Figure 5). During the recovery phase of the microbiota, we previously reported that subjects on the EEN diet showed a reduced recovery of GHs involved in the digestion of plant-based glycans,^18^ consistent with the predominance of taxa belonging to the Proteobacteria phylum relative to either Firmicutes or Bacteroidetes phyla, the latter of which are known to have a lower GH representation.^19^ The antibiotic and polyethylene glycol gut purge was associated with a substantial increase in fecal primary conjugated bile acids (GCDCA, TCDCA, GCA, and TCA), reflecting microbiota depletion and BSH loss, an effect which occurred in all diets (Day 9, Figure 5). Upon recovery of the gut microbiota from days 9–15, the fiber-containing vegan and omnivore diets were associated with rapid reduction of fecal primary conjugated bile acids, indicating the recovery of BSH activity. However, this recovery was not seen in those patients receiving the fiber-free EEN diet (Figure 5). The results from FARMM show that dietary fiber is associated with BSH activity in the human gut microbiome, mirroring the results in the SBS population. Indeed, the adult EEN intervention group from the FARMM study serves as a compelling model for children with short bowel syndrome who are nutritionally maintained on fiber-free EEN or PN, and who receive antibiotic therapy for suspected SIBO.

### Dietary fiber induces the deconjugation of primary bile acids specifically in the small intestine leading to increased plasma levels

**Figure 6.**
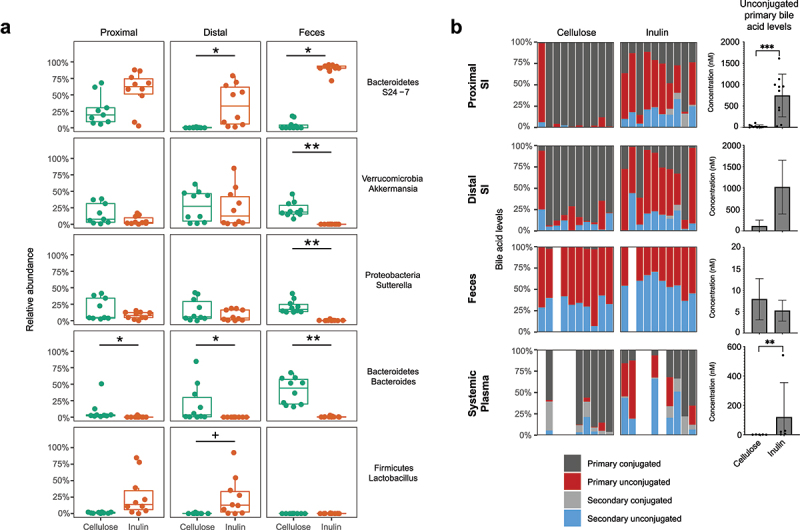
Comparison of intestinal bile acid levels throughout the length of the murine gut as well in systemic plasma between mice fed a non-fermentable cellulose vs. a fermentable inulin containing diet. (a) Differentially abundant taxa in the proximal small intestine, distal small intestine, and feces of mice fed a cellulose or inulin containing diet (linear mixed effects modeling with log transformed relative abundances; +q < 0.1; *q < 0.05, **q < 0.01). (b) Bile acid profiles and unconjugated primary bile acid levels in the proximal small intestine, distal small intestine, feces, and systemic plasma of mice fed a cellulose or inulin containing diet (unconjugated primary bile acids tested using Mann-Whitney tests; * p < .05, ** p < .01, *** p < .001).

Primary bile acids are normally deconjugated by BSH with subsequent conversion to secondary bile acids in the colon. However, when BSH function is induced proximally in the small intestine, as with small intestinal bacterial overgrowth, the subsequent deconjugation of primary bile acids reduced nutrient absorption leading to fat malabsorption.^27^ Since the regional effects of BSH activity are difficult to assess in the human small bowel, we developed a murine model system to determine the effect of fiber on the conjugation status of primary bile acids, specifically in the small intestine. Mice fed a diet containing inulin, a fermentable fiber, relative to those fed cellulose, a nonfermentable fiber, demonstrated significant reduction in alpha-diversity specifically in the proximal small intestine (Supplemental Figure 4A). There were also alterations in the composition of the gut microbiota throughout the length of the gut in both an unweighted and weighted Unifrac analysis (Supplemental Figures 4B and C, respectively). Inulin induced both *Muribaculum spp*, which are known to have high genomic representation of GHs, as well as *Lactobacilli spp*, which are known to have high representation of BSH genes^3,28,29^ (Figure 6a). Consistent with the induction of *Lactobacilli spp*. specifically in the small intestine, quantitative profiling of bile acids revealed that inulin led to the deconjugation of primary bile acids specifically in the small intestine (Figure 6b and Supplemental Figure 4D). Additionally, total bile acids levels were elevated in the small intestine, but not the feces (Supplemental Figure 4D). Plasma levels of unconjugated primary bile acids were elevated in mice fed inulin (Figure 6b and Supplemental Figure 4D), consistent with the increase in water solubility and absorption of deconjugated bile acids in the small intestine. This profile was also observed in children with SBS relative to healthy control children (Figure 3c) suggesting that plasma levels of unconjugated bile acids may have utility as a biomarker for SIBO and BSH activity in the small intestine.

## Discussion

Although most gut microbiota studies focus on colonic communities via the analysis of fecal samples, there is growing recognition for the importance of the small intestinal microbiota. Murine model systems suggest that the small intestinal microbiota may play a role in obesity, metabolic syndrome,^30^ celiac disease,^31^ and the engraftment of probiotics^32^ to name a few. The best characterized diseases involving the small intestinal microbiota in humans are SIBO and environmental enteric dysfunction^33^ where an alteration in the composition and/or biomass of the small intestinal microbiota play a role in disease pathogenesis. Unlike the diverse and relatively stable colonic microbiota, the small intestinal microbiota is believed to have lower diversity, and involves sub-daily fluctuations due to its exposure to dietary nutrients, yet there are few studies indicating the importance of this interaction on disease pathogenesis in humans.^34^

Herein, we characterize the effect of diet on the gut microbiota in patients with SBS. Since some patients with SBS have difficulty consuming a whole food diet due to symptoms, and thus, difficulty maintaining adequate nutritional intake, it is assumed that the gut microbiota contributes to these features. However, evidence to support this notion is lacking. We show not only that the composition of the gut microbiota in children with SBS is different from that in health but the variety of diets being consumed by these patients, ranging from 100% of calories from PN to 100% of calories from whole food intake, provided us with a unique opportunity to make correlations between diet and the microbiome.

The gut microbiota in children with SBS had lower a-diversity,^17,35,36^ lower Bacteroidetes, and greater abundance of Proteobacteria relative to healthy controls – a pattern correlated with the use of PN. However, the increasing consumption of whole food is correlated with higher levels of Bacteroidetes belonging primarily to the Bacteroides genera, and a decrease in Proteobacteria, primarily Enterobacteriaceae. The strong positive correlation between percent whole food intake and the relative abundance of GHs suggests that dietary fiber may be the major component of a whole food diet that is regulating gut microbiome composition in SBS patients. This would be consistent with the increase in *Bacteroides spp*. known to be rich in enzyme systems such as glycoside hydrolases needed for plant fiber degradation.^9^ Our findings that dietary regimen is the most significant factor impacting the microbiota in SBS contrasts with previous studies reporting that small bowel length had the largest impact on the microbiota in SBS.^16,37^

Compositional profiling of fecal bile acids in these same patients led us to consider a previously unrecognized association between GHs and (BSHs), thereby connecting two fundamental properties of gut microbiota function, glycan degradation, and bile acid metabolism. Whole food intake was positively correlated with 15 GH genes, which encode for enzymes that degrade complex polysaccharides, whereas the GHs involved in processing of simple carbohydrates were negatively correlated with whole food intake and more highly expressed in subjects consuming fiber-free diets (e.g., PN and/or EN). These results are consistent with our previous dietary intervention study of healthy adult subjects,^18^ which found that consumption of a fiber-free diet shifts the human gut microbiome away from complex glycan degradation and toward metabolism of simple carbohydrates.^18^

Importantly, we found that GHs targeting complex glycans found in plants were positively correlated with BSHs, which are widely distributed amongst the four major bacterial Phyla of the gut microbiome with proportionally lower abundance in Proteobacteria.^29^ The functional consequence of this correlation is supported by several observations in our study. First, the consumption of whole food was negatively correlated with the fecal abundance of conjugated bile acids. Second, the fecal bile acids in children with SBS were mostly unconjugated primary bile acids, compared to healthy children with predominantly secondary unconjugated bile acids. The high levels of fecal bile acids in these patients are due to the surgical resection of the terminal ileum, leading to both a reduction in reabsorption and reduced inhibition of hepatic bile acid synthesis, the latter supported by a reduction in plasma FGF19 levels. Third, we show in our FARMM study that a fiber-free EEN diet fails to support recovery of bacteria with BSH activity after depletion by an antibiotic/PEG gut purge and thus prevents the deconjugation of primary bile acids upon the recovery of the gut microbiota after depletion by an antibiotic/PEG gut purge. Finally, consumption of dietary fiber in the form of inulin by mice leads to an increase in bacteria genera known to have BSH activity, *Muribaculum*^38^ and *Lactobacillus*,^39^ concurrent with an increase in the deconjugation of primary bile acids, specifically in the small intestine. The localization of fiber-dependent deconjugation of primary bile acids to the small intestine is of significant physiological importance as it phenocopies the clinical consequences of SIBO in patients with SBS, not only with symptoms of gas, bloating, and abdominal pain, but also malabsorption. In total, exacerbation of SIBO with the introduction of whole foods containing dietary fiber may play a significant role in food intolerance in patients with SBS by increasing bacterial taxa with GH activity and augmenting BSHs, leading to the deconjugation of primary bile acids in the small intestine and exacerbating malabsorption.

The high levels of fecal unconjugated primary bile acids in patients with SBS is consistent with the lack of BAI operon activity in the gut microbiome to dehydroxylate unconjugated primary bile acids to generate secondary bile acids. An increase in unconjugated bile acids would also have an effect on their systemic absorption. Indeed, we also show that the dietary fiber-dependent deconjugation of primary bile acids in the small intestine of mice led to increased absorption of unconjugated primary bile acids with higher levels in systemic circulation. Since plasma levels of unconjugated primary bile acids are also increased in patients with SBS,^36^ plasma bile acid profiling might have utility as a modality to diagnose SIBO in patients with SBS, where typical diagnostic modalities like hydrogen breath testing and duodenal aspirate is of limited utility due to altered anatomy and motility.

The inherent challenges of caring for children with SBS with suspected SIBO also affords opportunity to expand diagnostic platforms. Integrating microbiome analytics with a focus on GH and BSH gene analysis, together with the characterization of fecal and plasma bile acids levels, provides insight into bacterial mechanisms relevant to clinical outcomes that are important for diet and nutrition. Through the use of Gaussian mixture modeling, we show that patients can be divided into one of three groups based on the composition of fecal bile acids – Partial Deconjugation, Complete Deconjugation, and Deconjugation/Dehydroxylation. Since these classifications are highly correlated with percent whole food intake, they may have value in predicting tolerance to dietary intake where fecal and plasma bile acid assays could be used to monitor therapeutic side effects.

Given the cross-sectional nature of this study analysis, our findings cannot be extrapolated to predict whole food tolerance and monitor side effects. Further prospective sampling will build on the current proposed classification to answer these questions. However, if validated, our findings may have utility in the development of more personalized therapeutic approaches focused on the gut microbiota and bile acid physiology. For example, patients whose main biological disturbance is robust deconjugation of primary bile acids may benefit from a BSH inhibitor^40^ or cholylsarcosine,^41^ whereas those patients who do not convert primary to secondary bile acids may benefit from bile acid binding resins like cholestyramine or targeted engraftment of *Clostridia* species.^42^ Indeed, our findings provide evidence for the potential value of the gut microbiome and its co-metabolites as a prognostic platform for precision nutrition and other therapeutic strategies in patients with SBS. Such a paradigm may have relevance to other diseases such as intestinal motility disorders associated with SIBO and environmental enteric dysfunction where alterations in the small intestinal microbiota have been described.^33^

## Methods

### Participants and study design

This is a cross-sectional investigation of the gut microbiota and metabolome in pediatric patients with SBS who received care in the Intestinal Rehabilitation Program (IRP) at Children’s Hospital of Philadelphia (CHOP) (IRB# 17–01400). All enrolled participants gave informed consent and had a history of SBS based on surgical and imaging records, as well as a history of parenteral nutrition (PN) dependence beyond 2 months. Patients with an established primary dysmotility disorder, inflammatory bowel disease, malignancy, immunodeficiency and/or antibiotic exposure within two weeks of enrollment (other than for the clinical indication of suspected SIBO) were excluded. For the 55 patients enrolled, clinical data were abstracted from medical charts and/or interview. Nutritional data, including PN composition, enteral formula regimens, dietary recall of foods, and caloric intake were abstracted from dietitian assessments performed closest in time to biospecimen collections. Percent calorie intake was extracted for PN, formulas and whole foods. Caloric intake from whole foods included orally consumed whole foods, as well as blenderized formulas consisting of whole food components that are commercially available or homemade, which may be given orally or via feeding tube. Caloric intake from enteral nutrition (EN) included only conventional formulas that are devoid of fiber. Antibiotic use was defined as current (actively on antibiotics at time of sample collection or having completed a course of antibiotics within the prior 10 days), recent (having completed at least one course of antibiotics between 10 and 90 days prior to sample collection), and none (no antibiotic usage within 90 days of sample collection). Samples were also collected from healthy children recruited from general pediatric clinics at CHOP (IRB# 15–011817; n = 114; age 4.53 ± 3.69 years, mean ± SD; 50.9% female)^43^ as well as a separate study known as COMBO3 (IRB# 810009; n = 12; age 5.5 ± 3.99 years, mean ± SD; 58% female) performed at the CHOP Clinical and Translational Research Center. Together, the size of the healthy control cohort was 126 (51.6% female) with a mean age of 4.62 ± 3.73 years.

### Sample collection

Stool, urine, and plasma were collected at enrollment. Efforts were made to collect stool/ostomy and plasma samples in the office setting at the time of a clinic appointment. Alternatively, the family was given a kit of supplies with detailed instructions for collection at home and for return to the study team. When available, we analyzed fecal samples collected on the same day for each subject. For five patients where inadequate specimen was available for both shotgun metagenomic and metabolomics analyses, we used samples collected from the next closest date, while ensuring overall clinical status remained the same amongst samples analyzed for each subject. 46 stool samples were analyzed for microbiome composition, 45 stool samples were analyzed for bile acid composition, and 35 plasma samples were analyzed for bile acid composition and FGF19 levels.

### Shotgun metagenomic sequencing and analysis

Shotgun metagenomic sequencing was performed as previously described.^18^ Data were analyzed using Sunbeam.^44^ Additional information on sequencing and analysis is available in Supplemental Materials. The relative abundance of BSH and GHs were determined using simple linear regression in GraphPad Prism 9. Correlations were considered significant if the Benjamini-Hochberg-adjusted p-value was less than 0.05.

### Fecal and plasma metabolites

Plasma FGF19 levels were quantified using solid-phase sandwich ELISA according to the manufacturer’s instructions (Human FGF-19 Quantikine ELISA Cat# DF1900, R&D Systems Inc., Minneapolis, MN). Bile acids levels were quantified using uPLC-MS as previously described.^45^

### Mouse studies

All animal experiments were performed according to Institutional Animal Care and Use Committee-approved protocols at the University of Pennsylvania (IACUC protocol # 803408) and adhere to the Animal Research: Reporting of *In Vivo* Experiments (ARRIVE) standards. Pure-bred female C57Bl/6 J mice (Jackson Laboratories) were fed compositionally defined diets purchased from Research Diets containing either non-fermentable cellulose or fermentable inulin fiber (see supplemental information for exact formulations, Table S2). Systemic blood, small intestinal contents, and feces were collected on Day 14. Small intestinal contents and feces were sequenced, and bile acid levels were quantified in the systemic blood, small intestinal contents, and feces as previously described.^45^

## Supplementary Material

Supplemental Material

## Data Availability

The data that support the findings of this study are available in Mendeley Data at DOI: 10.17632/wg4bwm5np8.1 and at the SRA, reference number PLACEHOLDER.

## Abreviation

bile-acid-induced operon (BAI), bile salt hydrolases (BSH), exclusive enteral nutrition (EEN), farnesoid X receptor (FXR), fibroblast growth factor 19 (FGF19), glycoside hydrolase (GH), Intestinal Rehabilitation Program (IRP), parenteral nutrition (PN), short bowel syndrome (SBS), small intestinal bacterial overgrowth (SIBO), enteral nutrition (EN), glycochenodeoxycholic acid (GCDCA), Taurochenodeoxycholic acid (TCDCA), Glycocholic acid (GCA), Taurocholic acid (TCA), Chenodeoxycholic acid (CDCA), cholic acid (CA), Deoxycholic acid (DCA), Glycodeoxycholic acid (GDCA), glycolithocholic acid (GLCA).
