# Supplementary material for: Dietary fiber-based regulation of bile salt hydrolase activity in the gut microbiota and its relevance to human disease
Source: Gut Microbes. 2022 Jun 5;14(1):2083417. doi: 10.1080/19490976.2022.2083417 (PMC9176262; doi:10.1080/19490976.2022.2083417)
Supplement: Supplemental Material [file KGMI_A_2083417_SM0711.zip › MOTIFS_SupplementalMethods_V3.docx]

**Supplemental Methods**

*Shotgun Metagenomic Sequencing*

Stool was frozen at -80˚C until analysis. DNA was extracted from fecal swab samples shaken at 20 Hz for 10 minutes using Qiagen DNeasy PowerSoil Kit, eluting 80ul of DNA. DNA was then quantified using the Quant-iTTM PicoGreenTM dsDNA assay Kit (Thermo Fisher Scientific) before library generation. A 10-fold dilution of the DNA to 0.4 ng/ul was made for NexteraXT library preparation. Shotgun libraries were generated from the 0.4ng/ul DNA dilution using the Illumina NexteraXT Library Prep Kit and Illumina Nextera Index Kit (Set B). Library success was assessed by Quant-iTTM PicoGreenTM dsDNA assay after Ampure Cleanup of Shotgun libraries using SPRI/AMPureXP beads in 50% PEG 8000 solution. Samples with library yields < 1 ng/ul were re-prepped as needed. Prior to library re-prep, DNA was cleaned up using Qiagen DNeasy PowerClean Pro Cleanup Kit. Reprepped sample libraries were Qubited using Thermo Fisher Qubit HS DNA Assay Kit to confirm library success. Library quality control was assessed through Fragment Analysis using Advanced Analytical High Sensitivity NGS Fragment Kit. All library samples were then pooled at an equal volume. The library pool was QCed on the Agilent BioAnalyzer to check the size distribution and absence of additional adaptor fragments. This QC pool was then sequenced using a 300 cycle Nano Kit on the Illumina MiSeq. Libraries were then repooled based on the demultiplexing statistics of the MiSeq Nano run. Final repooled libraries went through cBot clustering using HiSeq PE Cluster Kit v4 Box 1 of 2 prior to high-throughput sequencing using 250 cycle HiSeq SBS Kit v4 Boxes 1 of 2 and 2 of 2 as well as HiSeq PE Cluster Kit v4 – cBot Box 2 of 2 on the Illumina HiSeq. Extraction blanks and nucleic acid-free water were processed to empirically assess environmental and reagent contamination. A laboratory-generated mock community consisting of DNA from *Vibrio campbellii* and Lambda phage were included as a positive sequencing control.

*Bioinformatic Analyses:*

Shotgun metagenomic data were analyzed using Sunbeam^1^. The low-quality reads from fecal and rectal swab samples were discarded from further analysis using Trimmomatic-0.33^2^. Reads mapping to the human (version hg38) and PhiX genomes were filtered using BWA^3^. The abundance of bacteria was estimated using Kraken^4^. Linear models were used to estimate mean difference between clinical variables. Alpha diversity was calculated using Shannon diversity and the number of unique organisms observed per 1000 reads. Either alpha diversity or the logit transformed relative abundance of bacteria were used as the outcome variable, the clinical variable was used as a fixed effect, and any antibiotic use was used as a covariate. For categorical variables, Tukey post-hoc tests were done to estimate the difference between pairs of groups.

Reads were mapped to the KEGG database of bacterial proteins^5^, as well as curated databases of genes involved in bile acid production using Diamond search^6^. The alignments to the KEGG database were then mapped to gene orthologs and enzyme commission (EC) numbers. Glycoside hydrolase genes were identified as the reads that mapped to EC:3.2.1 category. Sample similarity was assessed by Bray-Curtis and Jaccard distances, and community-level differences between sample groups were assessed using the PERMANOVA test. P-values from multiple testing procedures were corrected to control for a specified false discovery rate using Benjamini-Hochberg method.

*Animal Experiment*

All animal experiments were performed according to Institutional Animal Care and Use Committee-approved protocols. Pure-bred female C57Bl/6J mice (Jackson Laboratories) at 12-14 weeks of age were housed in small-barrier cages (3-5 per cage), and maintained on standard vivarium chow (LabDiet 5053, Lab Supply, Fort Worth, TX) until the start of the two-week dietary intervention. A control group was continued on vivarium chow to establish normal values for the longitudinal assessment of metabolites in the lumen and circulatory compartments.

Mice were fed compositionally defined diets purchased from Research Diets (see supplemental information for exact formulations, **Table S2**). Both compositionally defined purified diets were similar in macronutrient breakdown to vivarium chow. Carbohydrate was provided in the form of corn starch and maltodextrin. The diets were irradiated and prepared with either non-fermentable cellulose, or fermentable inulin fiber. Based upon prior published work, 200g of inulin was chosen for the fermentable fiber.^7^ Mice had ad libitum access to assigned diet. The amount of diet consumed was determined per cage and averaged over the number of animals per cage. Weights and fecal pellets were collected at baseline (day 0), day 7, and day 14.

Submandibular cheek bleeds were performed to collect blood samples at baseline. On day 14, mice were anesthetized using isoflurane, and the portal vein accessed via abdominal cavity to collect 200-300 microliters of blood. The remainder of the blood volume was recovered via cardiac puncture. Blood was collected in lithium heparin tubes, centrifuged at 3,000 rpm for 10 minutes, and then plasma stored at -80^0^C. The liver, colon, and small bowel were then quickly harvested and stored at -80^0^C. To collect the small bowel luminal contents, the small bowel was divided equally, each half then flushed using a gavage needle with 1cc PBS. Small bowel flushes were then homogenized and centrifuged, and the supernatant was stored at -80^0^C. A 1cm section of each of the duodenum, mid-jejunum, and terminal ileum were placed in RNAlater and stored at -80^0^C.

*16S rRNA Gene Sequencing*

DNA was extracted direct from lysed fecal samples shaken at 20 Hz for 10 minutes using Qiagen DNeasy PowerSoil Kit, eluting 80ul of DNA. DNA was then quantified using the Quant-iTTM PicoGreenTM dsDNA assay Kit (Thermo Fisher Scientific) before library generation. For library generation, PCR reactions were carried out in quadruplicate using Q5 High-Fidelity DNA Polymerase (NEB, Ipswich, MA). Each PCR reaction had a total volume of 35ul, 5ul of low biomass DNA, 13.75 ul of Master Mix (5ul of 5X Buffer, 0.5ul of 10mM dNTPs, 1.25ul of 5uM forward primer,0.17ul of Q5 Pol, and 6.83ul of DNA-free water), and 6.25ul of 2uM reverse primer.  Cycling conditions are as follows: 1 cycle of 98C for 1 m; 25 cycles of 98C for 10 s, 56 C for 20 S, and 72C for 20 sec; 1 cycle of 72C for 8 m. After amplification, quadruplicate PCR reactions were combined into one 96 well plate and cleaned using SPRI/AMPureXP beads in 50% PEG 8000 solution at a ratio of 1:.9 (00ul of pooled pcr reaction: 90ul of SPRI Beads). Library success was assessed by Quant-iTTM PicoGreenTM dsDNA assay, then all libraries were pooled to a relatively equal number of nanograms. The sequencing pool was Qubited using Thermo Fisher Qubit HS DNA Assay Kit. The sequencing pool was loaded onto the Illumina Miseq at a loading molarity of 8pM with 15% PhiX using a 500 cycle Miseq Kit. Extraction blanks and nucleic acid-free water were processed to empirically assess environmental and reagent contamination. A laboratory-generated mock community consisting of DNA from Vibrio campbellii and Lambda phage were included as a positive sequencing control.

*16S rRNA Gene Sequence Analysis*

16S rRNA gene sequence data was processed with QIIME2^8^ using default parameters. Read pairs were processed to identify amplicon sequence variants (ASVs) with DADA2^9^. Taxonomic assignments for each ASV was generated using Greengenes taxonomy^10^ using the naïve Bayes classifier implemented in scikit-bio^11^. A phylogenetic tree was inferred using MAFFT^12^. Weighted and unweighted UniFrac^13^ distances were calculated for each pair of samples for assessment of community similarity and generation of principal coordinate analysis (PCoA) plots. Statistical analyses for bacterial abundance difference were performed using linear mixed effects models, and p-values were corrected for multiple comparisons using the Benjamini and Hochberg method.

*Bile acid quantification*

Bile acids were analyzed as previously described^14^. Briefly, fecal samples were suspended in methanol (5 μL/mg stool), vortexed for 1 minute, and centrifuged twice at 13,000g for 5 minutes. The supernatant was transferred to a new tube and quantified using a Waters Acquity uPLC System with a Cortecs UPLC C-18+ 1.6 μm 2.1 x 50 mm column and a QDa single quadrupole mass detector. Plasma samples were mixed 1:3 with methanol, briefly vortexed, and centrifuged at 13,000g for 5 min. All reagents were mass spectrometry grade.

1. Clarke EL, Taylor LJ, Zhao C, et al. Sunbeam: an extensible pipeline for analyzing metagenomic sequencing experiments. Microbiome 2019;7:46.

2. Bolger AM, Lohse M, Usadel B. Trimmomatic: a flexible trimmer for Illumina sequence data. Bioinformatics 2014;30:2114-20.

3. Li H, Durbin R. Fast and accurate short read alignment with Burrows-Wheeler transform. Bioinformatics 2009;25:1754-60.

4. Wood DE, Salzberg SL. Kraken: ultrafast metagenomic sequence classification using exact alignments. Genome Biol 2014;15:R46.

5. Ogata H, Goto S, Sato K, et al. KEGG: Kyoto Encyclopedia of Genes and Genomes. Nucleic Acids Res 1999;27:29-34.

6. Buchfink B, Xie C, Huson DH. Fast and sensitive protein alignment using DIAMOND. Nat Methods 2015;12:59-60.

7. Zou J, Chassaing B, Singh V, et al. Fiber-Mediated Nourishment of Gut Microbiota Protects against Diet-Induced Obesity by Restoring IL-22-Mediated Colonic Health. Cell Host Microbe 2018;23:41-53 e4.

8. Bolyen E, Rideout JR, Dillon MR, et al. Reproducible, interactive, scalable and extensible microbiome data science using QIIME 2. Nat Biotechnol 2019;37:852-857.

9. Callahan BJ, McMurdie PJ, Rosen MJ, et al. DADA2: High-resolution sample inference from Illumina amplicon data. Nat Methods 2016;13:581-3.

10. McDonald D, Price MN, Goodrich J, et al. An improved Greengenes taxonomy with explicit ranks for ecological and evolutionary analyses of bacteria and archaea. ISME J 2012;6:610-8.

11. Bokulich NA, Kaehler BD, Rideout JR, et al. Optimizing taxonomic classification of marker-gene amplicon sequences with QIIME 2's q2-feature-classifier plugin. Microbiome 2018;6:90.

12. Katoh K, Standley DM. MAFFT multiple sequence alignment software version 7: improvements in performance and usability. Mol Biol Evol 2013;30:772-80.

13. Lozupone C, Knight R. UniFrac: a new phylogenetic method for comparing microbial communities. Appl Environ Microbiol 2005;71:8228-35.

14. Friedman ES, Li Y, Shen TD, et al. FXR-Dependent Modulation of the Human Small Intestinal Microbiome by the Bile Acid Derivative Obeticholic Acid. Gastroenterology 2018;155:1741-1752 e5.
