# Supplementary material for: Dietary fiber-based regulation of bile salt hydrolase activity in the gut microbiota and its relevance to human disease
Source: Gut Microbes. 2022 Jun 5;14(1):2083417. doi: 10.1080/19490976.2022.2083417 (PMC9176262; doi:10.1080/19490976.2022.2083417)
Supplement: Supplemental Material [file KGMI_A_2083417_SM0711.zip › TableS1_V2.pdf]

Redcap ID/Record #	Age (years)	Whole Food (%)	Gaussian Group	ICV Present	Antibiotics Exposure	Anastomosis Type	Plasma Collection Date	Sequencing Stool Collection Date	Metabolomic Stool Collection Date	Dietary Information Collection Date
1	15.1	100	Complete Deconjugation	No	No	Jejuncolic	2/7/18	2/7/18	2/7/18	2/7/18
2	14.9	64	Complete Deconjugation	Yes	Current	Small Bowel/Small Bowel (At least 35cm of ileum remaining)	2/13/18	2/16/18	2/16/18	2/13/18
3	6.7	51	Complete Deconjugation	No	No	Small Bowel/Small Bowel (At least 15cm of ileum remains)	2/13/18	3/27/18	3/27/18	3/27/18
4	5.5	25	Complete Deconjugation	No	Recent	Jejunocolic	4/24/18	3/20/18	3/20/18	3/20/18
6	7.4	0	Partial Deconjugation	Yes	Recent	Jejunostomy (unknown length ileum remains)	2/20/18	2/20/18	2/20/18	2/20/18
7	7.5	100	Complete Deconjugation	No	No	Ileocolic(unknown length ileum remains)	2/20/18	3/2/18	3/2/18	2/20/18
8	3	0	Partial Deconjugation	No	Current	Duodenocolic(unknown length ileum remains)	2/27/18	5/17/18	5/17/18	5/8/18
9	8.5	46	Complete Deconjugation	No	Current	Ileocolic (1.5cm of ileum remains)	2/27/18	2/27/18	2/27/18	2/27/18
10	4.3	0	Partial Deconjugation	No	Recent	Jejuncolic	2/27/18	2/27/18	2/27/18	2/27/18
12	7.3	100	Complete Deconjugation	Yes	No	Small Bowel/Small Bowel (unknown length ileum remains)	n/a	3/13/18	3/13/18	3/13/18
13	7.4	23	Complete Deconjugation	Yes	Current	Colostomy (unknown length ileum remains)	3/13/18	3/13/18	3/13/18	3/13/18
14	11.3	20	Complete Deconjugation	No	No	Jejunocolic	5/1/18	5/1/18	5/1/18	5/1/18
15	4	5	Partial Deconjugation	No	Current	Ileocolic(unknown length ileum remains)	3/20/18	7/10/18	1/15/19	1/15/19
16	6.5	100	Complete Deconjugation	No	No	Ileocolic (unknown length ileum remains)	3/23/18	1/8/19	1/8/19	2/6/19
17	11.3	41	Partial Deconjugation	No	Current	Ileocolic (33cm of ileum remains)	3/27/18	3/27/18	3/27/18	3/27/18
19	5.6	58	Partial Deconjugation	No	Current	Jejunocolic	4/10/18	4/10/18	4/10/18	4/10/18
20	10	63	Deconjugation/Dehydroxylation	No	No	Ileocolic (80 cm of small intestine (jejunum & proximal ileum))	4/10/18	4/18/18	4/18/18	4/10/18
21	7.9	9	Complete Deconjugation	No	Current	Jejunocolic	4/10/18	4/10/18	4/10/18	4/10/18
22	2.3	67	Complete Deconjugation	No	Recent	Small Bowel/Small Bowel(unknown length ileum remains)	4/17/18	6/12/18	6/12/18	6/12/18
23	7.6	100	Complete Deconjugation	Yes	No	Small Bowel/Small Bowel(unknown length ileum remains)	8/14/18	12/14/18	12/14/18	12/14/18
24	10.1	73	Deconjugation/Dehydroxylation	Yes	Recent	Small Bowel/Small Bowel(unknown length ileum remains)	4/17/18	1/29/19	1/29/19	1/29/19
25	7.1	51	Complete Deconjugation	No	No	Ileocolic(unknown length ileum remains)	4/24/18	6/5/18	6/5/18	6/5/18
26	10.4	74	Complete Deconjugation	No	Current	Jejunocolic	n/a	5/29/18	5/29/18	5/29/18
27	0.5	10		No	Recent	Jejunocolic	6/20/18	6/20/18	10/24/18	6/20/18
28	1.6	16	Partial Deconjugation	No	Current	Jejunocolic	5/30/18	6/27/18	10/3/18	6/27/18
31	4.6	100	Deconjugation/Dehydroxylation	No	Recent	Ileocolic (unknown length ileum remains)	6/19/18	6/29/18	6/29/18	6/19/18
32	6.1	0	Deconjugation/Dehydroxylation	Yes	No	Small Bowel/Small Bowel(unknown length ileum remains)	n/a	7/18/18	7/28/18	6/19/18
33	0.6	12	Partial Deconjugation	Yes	No	Small Bowel/Small Bowel(unknown length ileum remains)	n/a	6/26/18	6/26/18	6/26/18
34	8.6	41	Complete Deconjugation	No	Current	Jejunocolic	6/27/18	6/27/18	6/27/18	6/27/18
35	3.6	61	Complete Deconjugation	No	No	Jejunocolic	10/23/18	10/23/18	7/24/18	10/23/18
36	14.7	100	Complete Deconjugation	No	Current	Ileocolic(unknown length ileum remains)	7/24/18	7/24/18	7/24/18	7/24/18
37	2.5	81	Complete Deconjugation	No	No	Ileocolic (left with 53 cm of small bowel (terminal ileum and ICV resected and ileum anastomosed to R colon)	n/a	10/2/18	10/2/18	10/2/18
38	3.8	88	Complete Deconjugation	No	No	Ileocolic(unknown length ileum remains)	n/a	7/31/18	7/31/18	7/31/18
39	3.5	40	Complete Deconjugation	No	Recent	Ileocolic(unknown length ileum remains)	n/a	8/3/18	8/3/18	7/25/18
40	1.5	0	Partial Deconjugation	No	Recent	Jejunostomy (unknown length ileum remains)	n/a	8/9/18	8/9/18	8/9/18
41	3.5	5	Complete Deconjugation	No	Recent	Ileostomy(unknown length ileum remains)	n/a	8/28/18	8/28/18	8/28/18
42	0.6	0	Complete Deconjugation	Yes	Recent	Small Bowel/Small Bowel (ileum was diminutive)	n/a	10/3/18	10/3/18	10/3/18
43	1.4	36	Partial Deconjugation	No	Recent	Jejunocolic	n/a	9/25/18	9/25/18	9/25/18
47	7.4	100	Complete Deconjugation	No	Current	Jejunocolic	11/9/18	12/13/18	12/13/18	12/13/18
48	0.6	0	Complete Deconjugation	Yes	No	Small Bowel/Small Bowel(unknown length ileum remains)	n/a	12/8/18	11/21/18	11/21/18
49	1	0	Partial Deconjugation	No	Current	Jejunostomy (at least 30cm ileal segment remains)	11/16/18	11/16/18	11/16/18	11/16/18
50	9.3	100	Deconjugation/Dehydroxylation	No	No	Ileocolic(unknown length ileum remains)	11/20/18	11/20/18	11/20/18	11/20/18
52	0.7	0	Partial Deconjugation	No	Recent	Ileocolic (unknown length ileum remains)	n/a	11/20/18	11/20/18	11/20/18
53	0.7	0	Partial Deconjugation	No	Recent	Jejunocolic	n/a	12/8/18	12/8/18	12/8/18
54	0.7	0	Partial Deconjugation	No	Current	Jejunocolic	12/4/18	12/4/18	12/4/18	12/4/18
55	5	28	Complete Deconjugation	No	No	Ileocolic(Hartmann pouch containing 7cm of distal ileum)	n/a	12/4/18	12/4/18	12/4/18
