# Supplementary material for: Dietary fiber-based regulation of bile salt hydrolase activity in the gut microbiota and its relevance to human disease
Source: Gut Microbes. 2022 Jun 5;14(1):2083417. doi: 10.1080/19490976.2022.2083417 (PMC9176262; doi:10.1080/19490976.2022.2083417)
Supplement: Supplemental Material [file KGMI_A_2083417_SM0711.zip › TableS2.pdf]

	Mass (g)														Mass (%)		
	Casein, 80 Mesh	L-Cystine	Corn Starch	Maltodextrin 10	Cellulose, BW200	Inulin	Flaxseed Oil	Mineral Mix S10026	Di-Calcium Phosphate	Calcium Carbonate	Potassium Citrate, 1 H_2O	Vitamin Mix V10001	Choline Bitarate	Total	Protein	Carbohydrate	Fat
**Cellulose**	200	3	575	125	50	0	45	10	13	5.5	16.5	10	2	1055	19%	67%	4%
**Inulin**	200	3	500	125	0	200	45	10	13	5.5	16.5	10	2	1130	18%	74%	4%

	Energy (kcal)														Energy (%)		
	Casein, 80 Mesh	L-Cystine	Corn Starch	Maltodextrin 10	Cellulose, BW200	Inulin	Flaxseed Oil	Mineral Mix S10026	Di-Calcium Phosphate	Calcium Carbonate	Potassium Citrate, 1 H_2O	Vitamin Mix V10001	Choline Bitarate	Total	Protein	Carbohydrate	Fat
**Cellulose**	800	12	2300	500	0	0	405	0	0	0	0	40	0	4057	20%	70%	10%
**Inulin**	800	12	2000	500	0	300	405	0	0	0	0	40	0	4057	20%	70%	10%
