# Supplementary figures and images for: Dietary fiber-based regulation of bile salt hydrolase activity in the gut microbiota and its relevance to human disease
Source: Gut Microbes. 2022 Jun 5;14(1):2083417. doi: 10.1080/19490976.2022.2083417 (PMC9176262; doi:10.1080/19490976.2022.2083417)

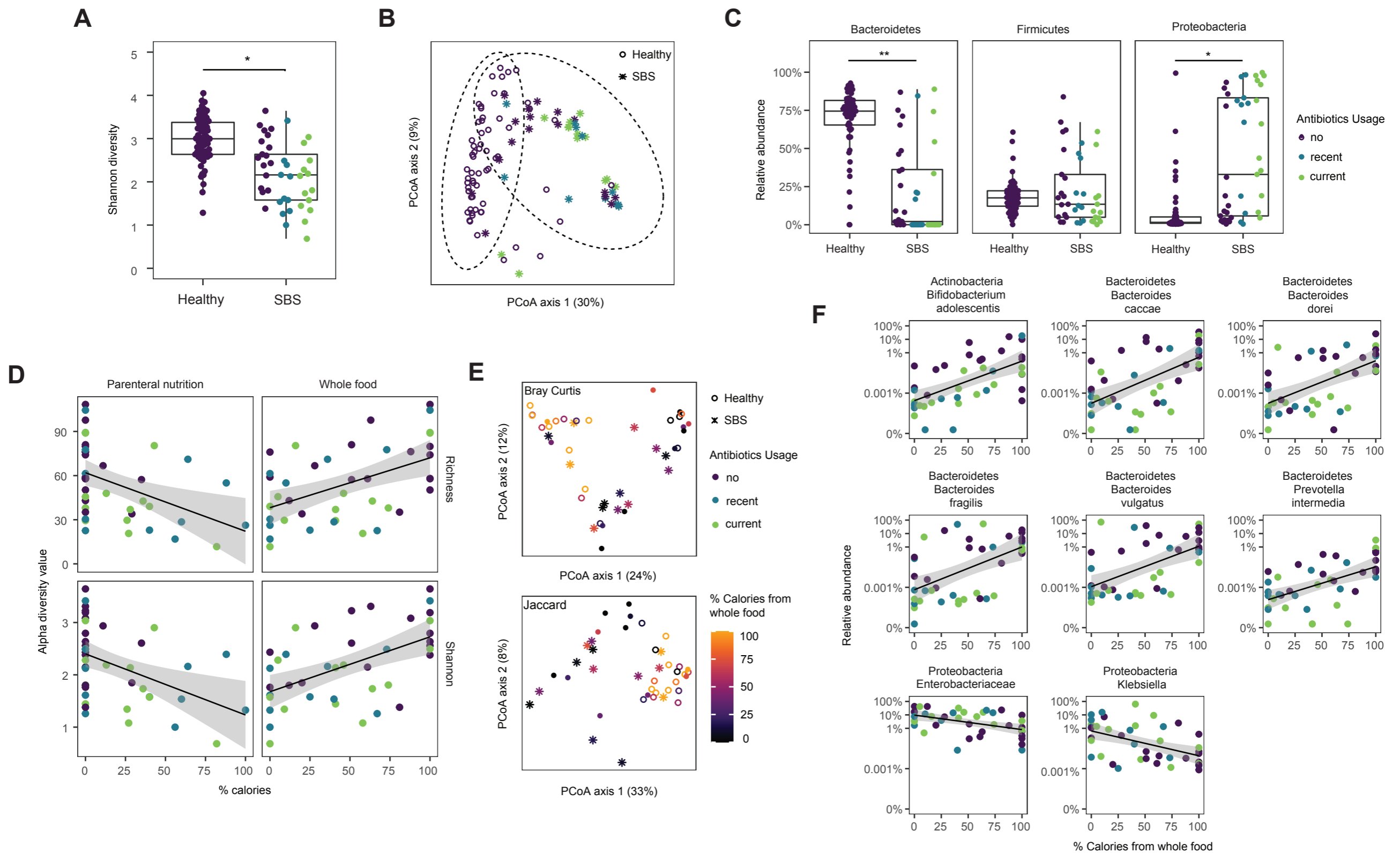

Figure 1

Supplement: Supplemental Material [file KGMI_A_2083417_SM0711.zip › SupplementalFigure1.pdf]

A

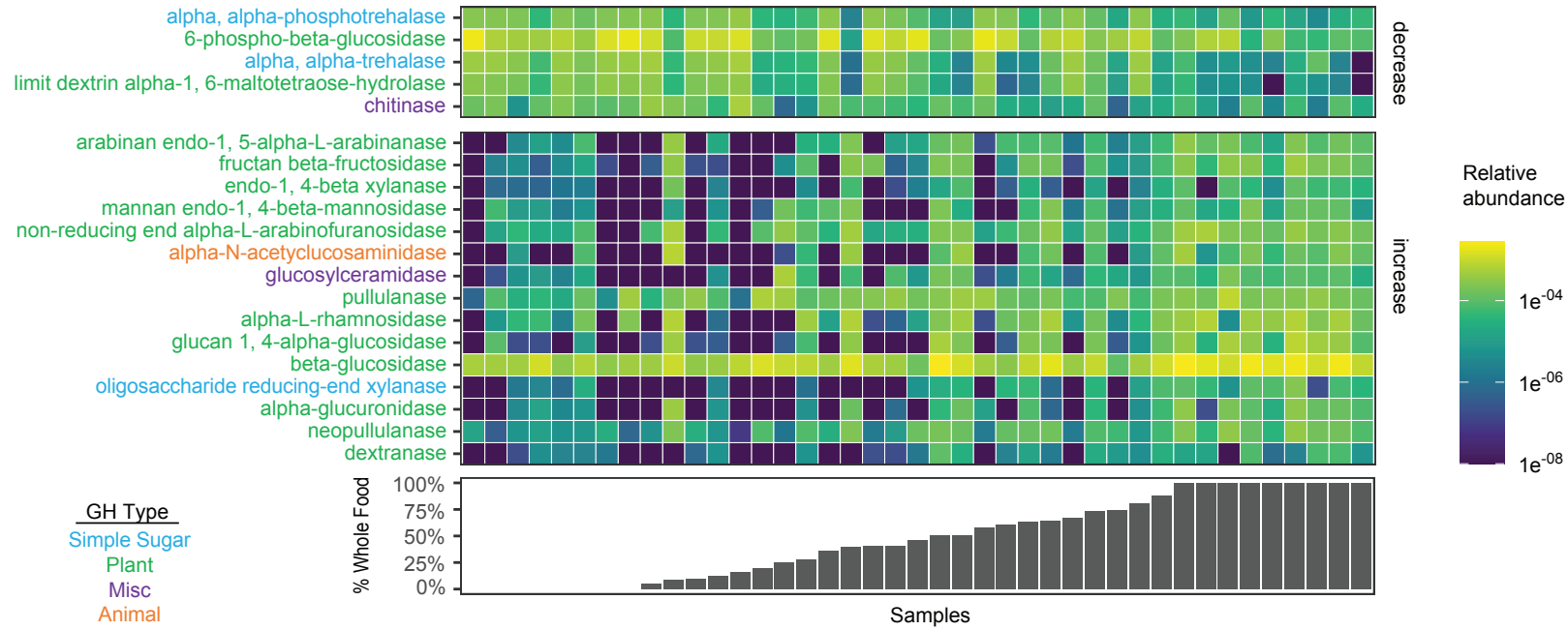

B

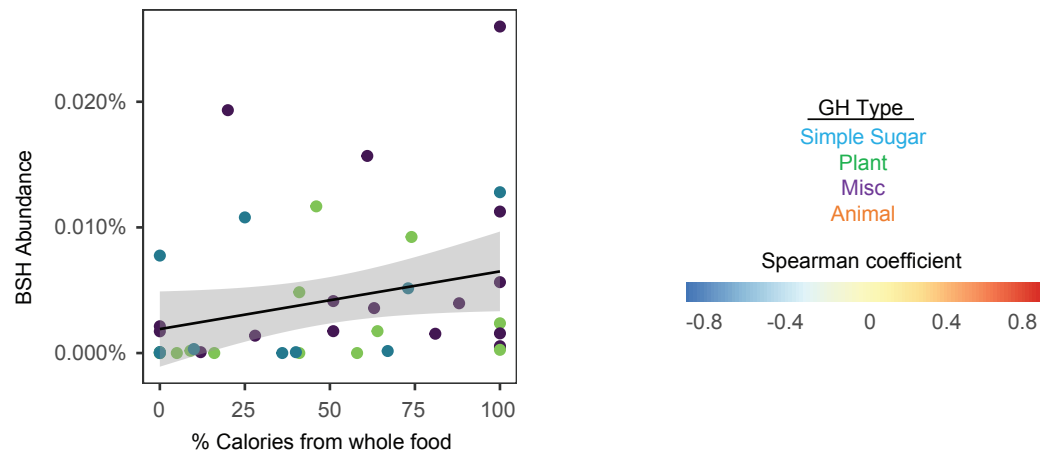

C

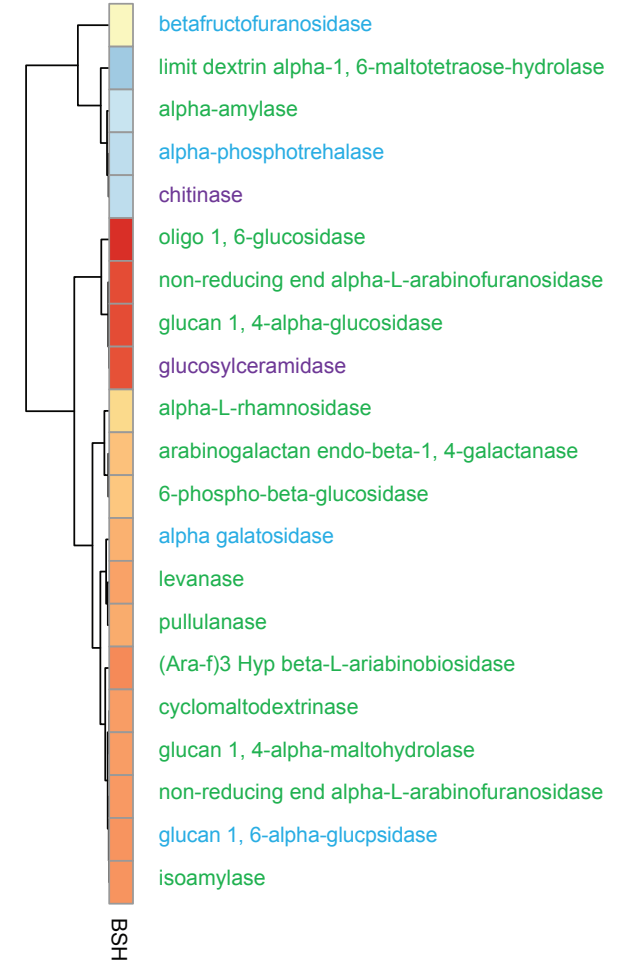

Figure 2

Supplement: Supplemental Material [file KGMI_A_2083417_SM0711.zip › SupplementalFigure2.pdf]

Relative abundance

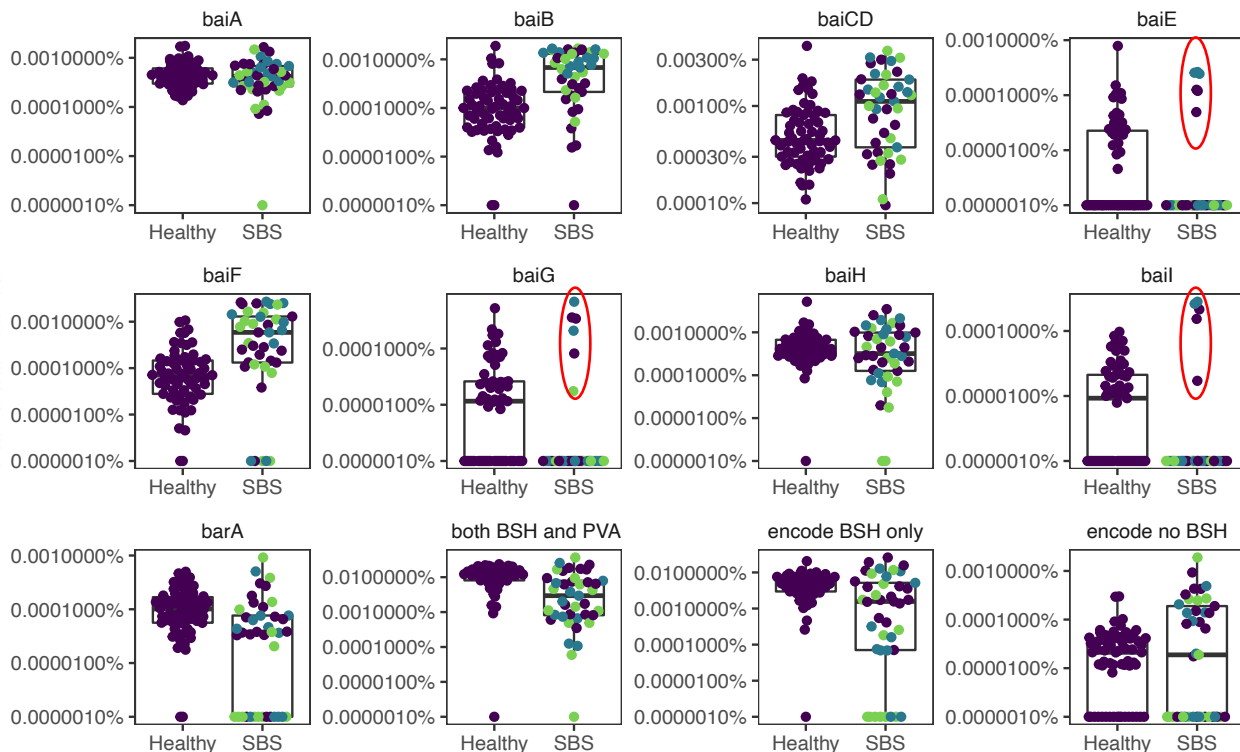

Abx use    no    recent    current

Supplemental Figure 3

Supplement: Supplemental Material [file KGMI_A_2083417_SM0711.zip › SupplementalFigure3 (1).pdf]

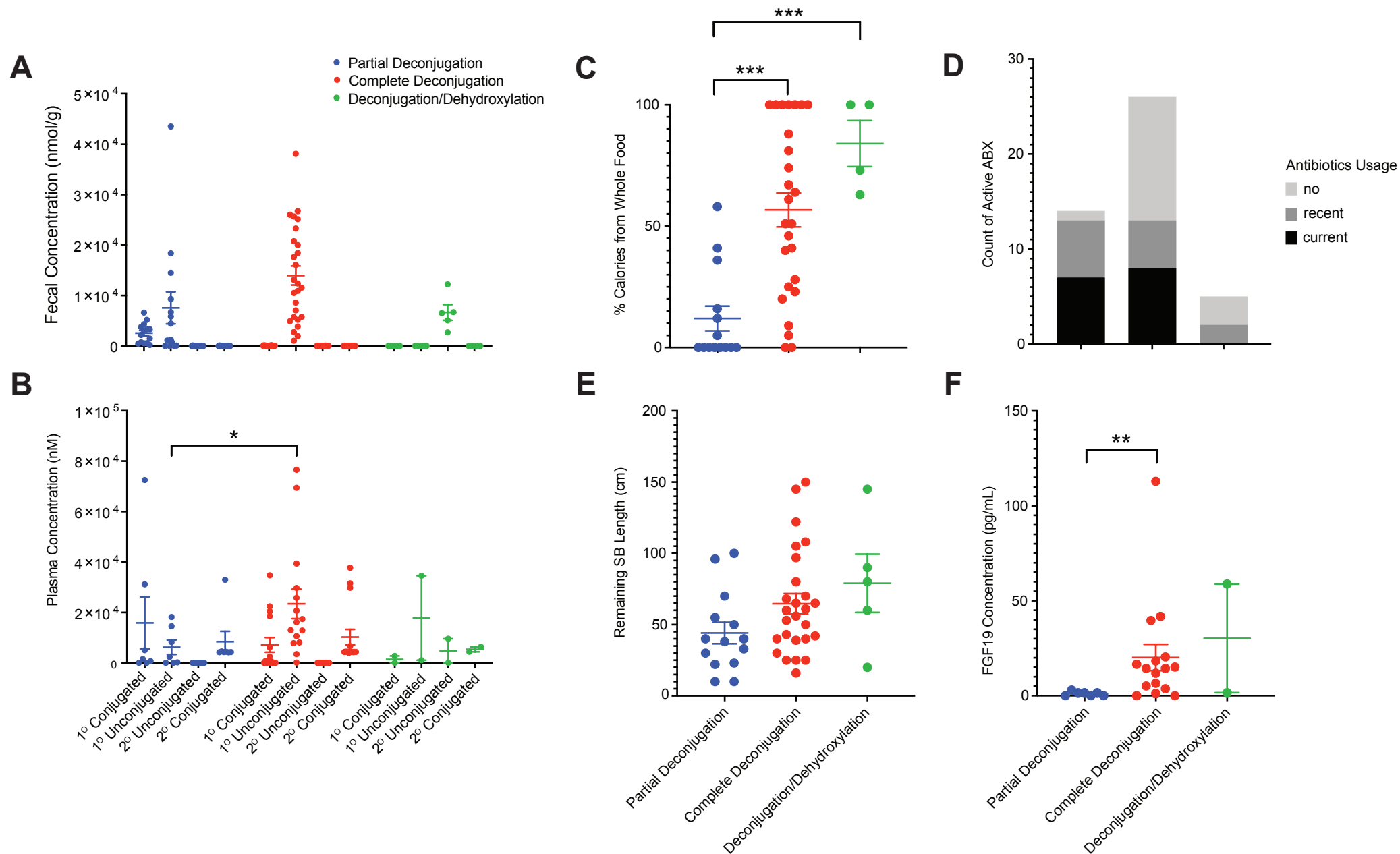

Figure 4

Supplement: Supplemental Material [file KGMI_A_2083417_SM0711.zip › SupplementalFigure4.pdf]
